# Transfusion transmissible infections among blood donors in Ghana: A 3‐year multicentered health facility‐based retrospective study

**DOI:** 10.1002/hsr2.1681

**Published:** 2023-11-01

**Authors:** Williams Walana, Ezekiel K. Vicar, Eugene D. Kuugbee, Isaac Dari, Grace Bichenlib, Christian N. Aneba, Kwasi N. Hinneh, Iddrisu B. Yabasin, Koray N. Issaka, Michael O. Danso, Theophilus N. Amoatey, Juventus B. Ziem

**Affiliations:** ^1^ Department of Clinical Microbiology School of Medicine, University for Development Studies Tamale Ghana; ^2^ Department of Microbiology and Immunology School of Medicine and Dentistry Navrongo Ghana; ^3^ Community Health and Preventive Medicine School of Medicine, University for Development Studies Tamale Ghana; ^4^ Department of Anaesthesiology and Intensive Care University for Development Studies Tamale Ghana; ^5^ Department of Laboratory Service Wa Municipal Hospital, Upper West Region Wa Ghana; ^6^ Department of Laboratory Service Weija‐Gbawe Municipal Hospital, Greater Accra Region Accra Ghana; ^7^ Nkwanta South Municipal Hospital, Oti Region Nkwantah Ghana

**Keywords:** HBV, HCV, HIV, Syphilis, *T. pallidum*, transfusion transmissible infections

## Abstract

Transfusion transmissible infections (TTIs) remain a major health challenge particularly in developing countries. Here, we present a multicentered hospital‐based retrospective study on the prevalence, distribution, and risk factors of TTIs in Ghana. Data on blood donors from four health facilities, namely Nkwanta South Municipal Hospital (Oti region), Weija‐Gbawe Municipal Hospital (Greater Accra region), SDA Hospital (Northern region) and Wa Municipal Hospital (Upper West region) were extracted and analyzed. Descriptive statistics and multinomial logistic regression were applied to compare sociodemographic data with TTI status. A total of 6094 blood donors were included in this study, and 2% were females. The overall prevalence of TTIs was 21.0% (1232/5868). Specifically, the prevalence of HBV, HCV, HIV, and Syphilis was 6.6% (385/5868), 4.9% (286/5830), 2.9% (168/5867), and 6.8% (393/5739), respectively. Wa dominated in all the viral agents considered in this study, while the Oti region recorded the highest prevalence in *T. pallidum*. The odds of HBV infection was 3.1 (*p* = 0.008) among first‐time donors, while that for HCV was 2.8 (*p* = 0.042). For rural dwellers, donors significantly had *T. pallidum* (*p* < 0.001; OR = 2.8), HCV (*p* < 0.001; OR = 2.9), and HIV (*p* = 0.028; OR = 1.5) infections. Generally, the recipients of transfused blood were predominantly pregnant mothers, followed by children and accident victims. This study has revealed significant disparities and relatively high prevalence of TTIs in Ghana, specifically HBV, HCV, HIV and *T. pallidum* infections. The variations suggest the presence of unique health challenges per study area, hence the need for a tailored intervention for each study site.

## 1 INTRODUCTION

Blood transfusion is undoubtedly an important medical intervention globally, saving millions of patients' lives. However, this life‐saving intervention is sometimes accompanied by transfusion transmissible infections (TTIs). Notably among these infections are the viral agents: hepatitis B virus (HBV), hepatitis C virus (HCV), and human immunodeficiency virus (HIV). The predominant bacteria cause of TTIs with significant medical importance is *Treponema pallidum*, the causative agent for syphilis.^1^

The burden posed by TTIs is multifaceted and more profound in developing countries than the industrialized ones. In the developing world, the prevalence of TTIs ranges from 1.03% to 3.70% and from 0.003% to 0.05% in the advanced economies.^2^ Specifically in Africa, TTIs associated with HIV ranges from 5% to 10%, whereas that of viral hepatitis due to posttransfusion infections is 12.5%, but *T. pallidum*‐associated TTI is relatively low.^3^, ^4^

Even though effective HBV prophylaxis has been available,^5^ there are ~2 billion infected people worldwide, with ~350 million persistently infected.^6^ HBV infects over 50 million people in Africa, with up to 20% of the infected persons in Sub‐Saharan Africa alone being chronic carriers.^6^ In Ghana, the prevalence of HBV infection ranges from 4.8% to 21%.^7^ Such chronic infections result in hepatitis‐related complications such as cirrhosis and liver carcinoma. For example, HBV infection is responsible for 59% of liver carcinomas identified in developing countries.^5^

It is estimated that 130–150 million people worldwide are afflicted with HCV, with Africa accounting for ~8% of all HCV infections worldwide.^8^, ^9^ Each year, around 500,000 people die due to HCV‐related liver illnesses.^10^ According to Holy et al.,^11^ in 2016, the prevalence of HCV among blood donors in Koforidua, Ghana, was 8.0%. Although HCV infections are common across West and North Africa, little attention is paid to them in Ghana,^12^ because acute HCV infection is typically asymptotic, and few patients are diagnosed accidentally during the acute phase.^13^

Since the onset of HIV, some 84.2 million people have been infected (ranging from 64.0 to 113.0 million victims) and the associated deaths have been pegged at 40.1 million.^14^ About 38.4 million people live with the virus and the global prevalence is 0.7%.^14^ The WHO African Region remains heavily impacted by HIV, with a significant proportion of its adult population, around 3.4% or ~1 in every 25 individuals living with the virus.^15^ Africa alone accounts for about two‐thirds of the global HIV population. In South Africa, the prevalence of HIV in Eswatini is 30.0% and that of Lesotho is 25.6%.^16^, ^17^ Ghana has a countrywide HIV prevalence of 1.6%, with regional rates varying; the Bono area has the highest regional HIV prevalence, at 2.5%.^18^

Syphilis is estimated to affect around 6 million individuals between the ages of 15 and 49 annually worldwide, leading to ~300,000 prenatal and neonatal deaths. Additionally, it increases the risk of early death in an additional 215,000 infants.^19^ In Africa, syphilis infection rates among women of reproductive age range from 0.36% to 3.6%, whereas blood donors have recorded rates ranging from 0.71% to 20%.^20^ A study conducted in Ghana revealed a prevalence rate of 0.3% among pregnant women and 3.7% among healthy blood donors).^21^

In Ghana, overall, the prevalence of TTIs with a focus on HBV, HCV, HIV, and *T. pallidum* was 11.8%, 8.3%, and 12.7% for the years 2014, 2015, and 2016 respectively.^22^ Blood donors in Ghana had an HIV prevalence of 2.6% and those with chronic hepatitis C were predicted to be 3.0%. The incidence varies noticeably between rural (5.7%) and urban (2.6%) environments.^23^ Some behavioral characteristics predispose to TTIs. HIV, HBV, HCV, and *T. pallidum* share common transmission routes and are linked to one's lifestyle. Men having sex with men, intravenous drug users, commercial sex workers, having multiple sex partners, and occupations particularly in the health sector, are among the risk factors.^24^

As the prevalence of these infections is spatially connected, it varies geographically. Aside the fact that there is limited data on TTIs in Ghana, most previous TTI studies are single‐centered and do not' reveal the geographical dispersions of TTIs in Ghana. In Ghana, as observed in many developing countries, the major source of blood for transfusion is from voluntary donors and replacement donors. In either way, the fundamental requirement must be the safety of the blood, which is premised on proper selection and screening of blood. To appreciate the risk of TTIs to the health of patients requiring blood transfusion in Ghana, it is important to establish the prevalence and the pattern of TTIs, particularly HBV, HCV, HIV, and *T. pallidum*, in a multicentered manner to guide local and national policy on the prevention and management of TTIs in Ghana, hence this study.

## 2 METHODS

### 2.1 Study sites

This multicentered study was conducted in four health facilities across four regions in Ghana. These included Nkwanta South Municipal Hospital (Oti region), Weija‐Gbawe Municipal Hospital (Greater Accra region), SDA Hospital (Northern region), and Wa Municipal Hospital (Upper West region). The Nkwanta South Municipality is geographically situated at 8°15′25.92″ N 0°31′6.96″ E, with a land surface area of 2733 km^2^, and about 75% of the population reside in rural areas that lack paved road infrastructure. The Weija‐Gbawe Municipality is in the southwestern part of Accra. It is situated at 5°33′59.76″ N 0°20′0.6″ W, with over 120 communities. Its land area is roughly 502.31 km^2^, home to about 213,674 people. The Wa Municipal forms part of the 11 municipalities and districts in the Upper West region. It is in the southeastern part of the region with Wa as its capital. It has a landmass of about 1078 km², which lies at 10°3′36.36″ N 2°30′35.64″ W. The Tamale municipality is one of the six Metropolitan Assemblies in the country and the only Metropolis in the Northern Region. It is situated at 9°24′30.1″ N 0°50′25.63″ W and located in the central part of the northern region, sharing boundaries with five other districts. The Metropolis had a population of ~701,000 in the year 2022.

### 2.2 Study design and data collection

This retrospective study spans the years 2020 to 2022. The study population included all individuals who visited the blood banks of the selected health facilities during the study period and were captured in the blood donor's register. These participants were first taken through the predonation education, donor history questionnaire, and then screened for hemoglobin level, and finally screened for TTIs. In the current study, all the health facilities screen donors using one‐step immune‐chromatographic assays available for HBV, HCV, HIV, and *T. pallidum*. None of the laboratories employed the enzyme‐linked immunosorbent assay or the polymerase chain reaction technique. After carefully studying the parameters in the blood donors' record books, a Google Form‐based questionnaire was developed and tested using data available in the record books. The questionnaire was reviewed after pretesting before actual data collection. The questionnaire captured data on “donors” demographics (which included age, sex, residence, frequency of blood donation, and type of blood donation) and status of TTIs, specifically for HBV, HCV, HIV, and *T. pallidum*. The sample for the study constituted all blood donors during the study period. In all, 6094 “donors” records were available, thus constituting the study's sample size.

### 2.3 Data analysis

The data from the Google Form was extracted and exported into IBM Statistical Package for Social Sciences (SPSS) software version 27 for analysis. The variables were predominantly categorical and the major demographic parameters captured were age, sex, residence of donor (rural or urban), and the frequency of blood donation. Multivariate analysis was performed to compare the demographic parameters with various TTIs and a measure of significant association was pegged at *p* < 0.05.

### 2.4 Ethical clearance

Approval for the study was granted by the University for Development Studies Institutional Review Board (UDS/RB/027/23). Permission was obtained from the hospital through the medical superintendents, the laboratory managers, and the head of blood bank services of the various health facilities. The data were collected anonymously to ensure confidentiality.

## 3 RESULTS

### 3.1 Demography of blood donors

Age‐wise, majority of blood donors were <35 years old and only about 2% of the donors were females. The proportion of the blood donors in the years under review showed slight variations. Health facilities with the highest number of blood donors' records was the Wa Municipal Hospital, followed by Nkwanta South Municipal Hospital. Blood donors came from both urban (50.6%) and rural (49.4%) dwellings. The donation types were predominantly family/friend donors (71.4%), replacement donors (25.4%), and volunteer donors (3.2%), as shown in Table 1.

**Table 1:** Demography features of blood donors.

Demographic features	Frequency	%
Age (years)		
≤23	1632	26.8
24–27	1130	18.6
28–34	1690	27.8
≥35	1633	26.8
Total	6085	100
Sex		
Female	122	2.0
Male	5970	98.0
Total	6092	100
Year	Frequency	
2020	2187	35.9
2021	1929	31.7
2022	1978	32.4
Total	6094	100
Hospital	Frequency	
Nkwanta South Municipal Hospital	1513	24.8
SDA Hospital, Tamale	251	4.2
Wa Municipal Hospital	3013	49.4
Weija‐Gbawe Municipal Hospital	1317	21.6
Total	6094	100
Residence	Frequency	
Rural	2968	49.4
Urban	3043	50.6
Total	6011	100
Type of donation	Frequency	
Family/friend donor	4211	71.4
Replacement	1496	25.4
Volunteer	187	3.2
Total	5894	100

### 3.2 Prevalence and distribution of TTIs among blood donors

In all, 1232 TTIs were recorded across the study sites, representing 21.0% (1232/5868) prevalence. The prevalence of TTIs based on blood donors from the Greater Accra, Oti, Northern, and Upper West regions were 11.5%, 31.2%, 1.3%, and 56.0%, respectively. Specifically with the infectious agents, the prevalence of HBV, HCV, HIV, and *T. pallidum* was accordingly 6.6% (385/5868), 4.9% (286/5830), 2.9% (168/5867), and 6.8% (393/5739). Per the study sites, the prevalence of HBV, HCV, HIV, and syphilis varied across the country. HBV prevalence was highest (7.5%) in Wa (in the Upper West region of Ghana), followed by 6.4% in Nkwanta South (in the Oti region of Ghana), and the least prevalence was 3.3% in Tamale. However, the prevalence of HCV in Wa was 7.7%, and that of Nkwanta South and Accra (Weija‐Gbawe Municipal Hospital) were 4.6% and 0.2%, respectively. Similarly, blood donors in Wa recorded the highest prevalence for HIV (5.1%) and 1.2% each for the Nkwanta South and Tamale. The overall prevalence for *T. pallidum* was 6.8%, Nkwanta South was 13.5%, and 5.2% for Weija‐Gbawe (Table 2).

**Table 2:** Prevalence and distribution of HBV, HCV, HIV, and syphilis.

Infection	Frequency (%)	Nkwanta South Municipal Hospital, *n* (%)	SDA Hospital Tamale, *n* (%)	Wa Municipal Hospital, *n* (%)	Weija‐Gbawe Municipal Hospital, *n* (%)
HBV					
Negative	5483 (93.4)	1395 (93.6)	238 (96.7)	2603 (92.5)	1247 (94.8)
Positive	385 (6.6)	96 (6.4)	8 (3.3)	212 (7.5)	69 (5.2)
Total	5868 (100.0)	1491 (100.0)	246 (100.0)	2815 (100.0)	1316 (100)
HCV					
Negative	5544 (95.1)	1422 (95.4)	240 (99.2)	2569 (92.3)	1313 (99.8)
Positive	286 (4.9)	68 (4.6)	2 (0.8)	214 (7.7)	2 (0.2)
Total	5830 (100.0)	1490 (100.0)	242 (100.0)	2783 (100.0)	1315 (100.0)
HIV					
Negative	5699 (97.1)	1471 (98.8)	242 (98.8)	2673 (94.9)	1313 (99.8)
Positive	168 (2.9)	18 (1.2)	3 (1.2)	144 (5.1)	3 (0.2)
Total	5867 (100.0)	1489 (100.0)	245 (100.0)	2817 (100.0)	1316 (100.0)
Syphilis					
Negative	5346 (93.2)	1297 (86.5)	243 (98.8)	2558 (95.5)	1248 (94.8)
Positive	393 (6.8)	202 (13.5)	3 (1.2)	120 (4.5)	68 (5.2)
Total	5739 (100.0)	1499 (100.0)	246 (100.0)	2678 (100.0)	1316 (100.0)
Total TTI	1232	384 (31.2)	16 (1.3)	690 (56.0)	142 (11.5)

Abbreviations: HBV, hepatitis B virus; HCV, hepatitis C virus; HIV, human immunodeficiency virus; TTI, transfusion transmissible infection.

### 3.3 Risk factors of TTIs among blood donors

From the multivariate analysis, the odds of HBV infection among blood donors were 2.5 and 2.0 for the years 2020 and 2021, respectively. Within the same period, HIV infection, although significant, had weaker odds of infection. Regarding the donors' age, HBV and HIV infections among the donors were not statistically significant. However, HCV infection (*p* = 0.016) was significant in donors <24 years, whereas syphilis infection was statistically significant among donors within the age group <24 years (*p* = 0.003) and 24–27 years (*p* = 0.022). The odds of HBV infection was 3.1 (*p* = 0.008) for first time donors, whereas that for HCV was 2.8 (*p* = 0.042). For *T. pallidum*, infection was significant (*p* = 0.022) among frequent blood donors. For rural dwellers, donors significantly had *T. pallidum* infection (*p* < 0.001; odds ratio = 2.8), whereas the odds for HCV infection among them was 2.9 (*p* < 0.001) and that of HIV was 1.5 (*p* = 0.028) (Table 3).

**Table 3:** Association between demography and TTIs among blood donors.

	HBV		HCV		HIV		Syphilis	
	*p*	AOR (95% CI)	*p*	AOR (95% CI)	*p*	AOR (95% CI)	*p*	AOR (95% CI)
Year								
2020	<0.001	2.5 (1.9–3.4)	0.816	1.0 (0.7–1.3)	<0.001	0.5 (0.3–0.7)	0.399	1.1 (0.9–1.4)
2021	<0.001	2.0 (1.5–2.7)	0.028	0.7 (0.5–1.0)	0.002	0.6 (0.4–0.8)	0.040	0.8 (0.6–1.0)
2022 (ref)								
Donors' age, years (ref)								
≤23	0.175	0.8 (0.6–1.1)	0.016	0.6 (0.5–0.9)	0.817	1.0 (0.6–1.5)	0.003	0.7 (0.5–0.9)
24–27	0.581	1.1 (0.8–1.5)	0.066	1.4 (1.0–2.0)	0.652	1.1 (0.7–1.7)	0.022	0.7 (0.5–0.9)
28–34	0.730	1.1 (0.8–1.4)	0.731	1.1 (0.8–1.5)	0.206	0.8 (0.5–1.2)	0.593	0.9 (0.7–1.2)
≥35 (ref)								
Frequency of donation								
First time donor	0.008	3.1 (1.3–7.0)	0.042	2.8 (1.0–7.7)	0.449	0.8 (0.4–1.6)	0.221	1.5 (0.8–2.9)
Second time donor	0.383	1.5 (0.6–3.8)	0.157	2.2 (0.7–6.6)	0.639	0.8 (0.3–1.9)	0.650	1.2 (0.6–2.5)
Frequent donors	0.539	0.7 (0.3–2.0)	0.306	1.8 (0.6–5.5)	0.181	0.5 (0.2–1.4)	0.022	0.3 (0.1–0.9)
Third time donor (ref)								
Resident of donors								
Rural	0.108	1.2 (1.0–1.5)	<0.001	2.9 (2.2–3.9)	0.028	1.5 (1.0–2.0)	<0.001	2.8 (2.2–3.5)
Urban (ref)								

Abbreviations: AOR, adjusted odds ratio; CI, confidence interval; HBV, hepatitis B virus; HCV, hepatitis C virus; HIV, human immunodeficiency virus; ref, reference category; TTI, transfusion transmissible infection.

### 3.4 TTI pattern, blood recipients, and blood‐type distribution at the study sites

**Figure 1:**
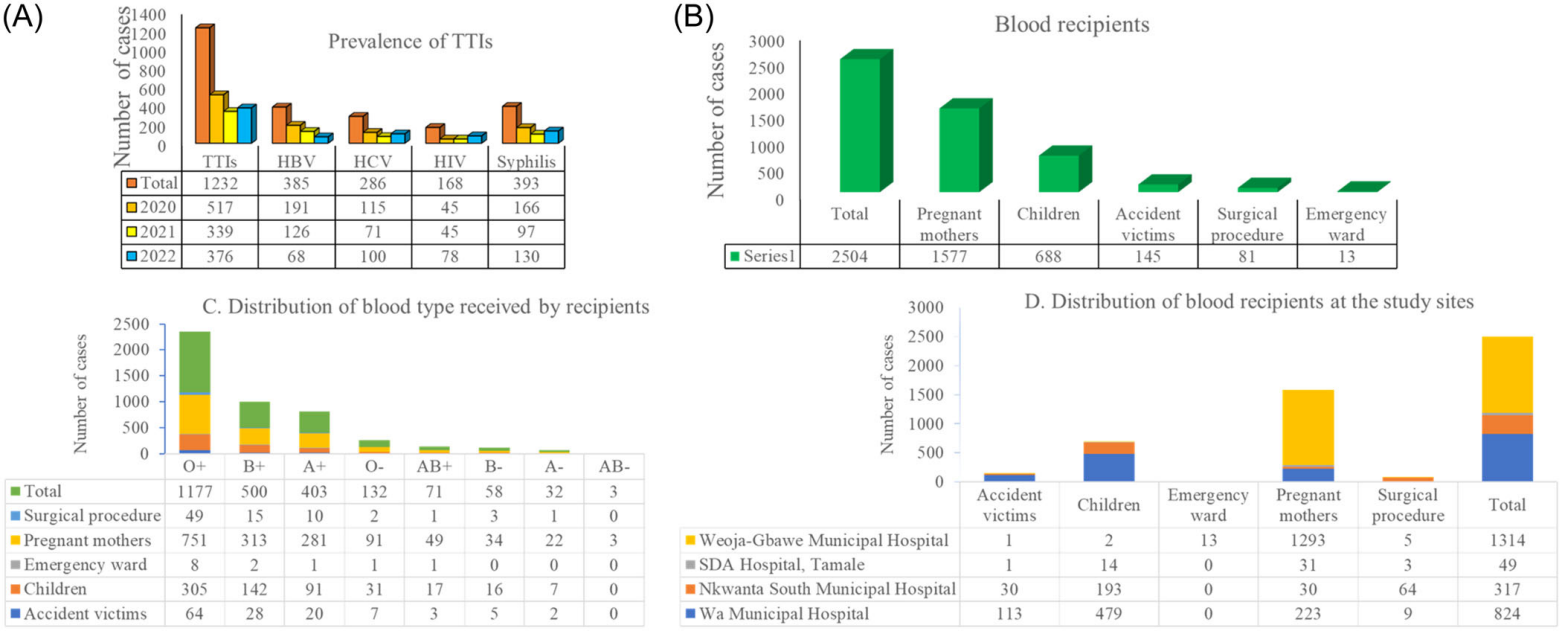
Transfusion transmissible infection (TTI) prevalence, blood recipients and blood‐type distribution at the study sites.

Trend‐wise, all the TTIs studied saw a downward trend from the year 2020 to 2021 but surged sharply in 2022, except for HBV (Figure 1A). The targeted blood recipients captured were predominantly pregnant mothers, followed by children, accident victims, surgical procedures, and emergency ward patients (Figure 1B). The pattern of blood types was principally O+, then B+ and A+, and the least blood type was AB− (Figure 1C). In Accra (Weija‐Gbawe Municipal Hospital), targeted blood recipients were hugely pregnant mothers as in Tamale, whereas in Wa (Wa Municipal Hospital) the major group of blood recipients were children, pregnant mothers, and accident victims. Similarly, in Nkwanta South (in the Oti region), children were the most recipients of blood transfusion, followed by surgical procedure (Figure 1D).

## 4 DISCUSSION

In this current multicentered study conducted in Ghana, the overall prevalence of TTIs among blood donors was 21.0%. This is comparable to similar single‐centered studies; 19.1% in Offinso‐North District in the central belt of Ghana,^25^ 19.3% in Nigeria, 24.0% in Central African Republic,^26^ but higher than 10.1% reported in northern Tanzania,^27^ 9.4% in Kenya,^28^ 3.4%–11.5% in Ethiopia,^29^, ^30^ and 3.6% in Eritrea.^31^ In a multicentered study among prison inmates in Ghana, the prevalence was about 25.5%.^32^ The prevalence observed in this study is far higher if compared with that of a similar study conducted in the United States, where prevalence was reported per 100,000.^33^

Specifically, the prevalence of HBV infection ranged from 3.3% in Tamale to 7.5% in Wa, with an overall prevalence of 6.6%. HCV overall prevalence was 4.9%, with 0.2% in Accra and 7.7% in Wa. Similarly, HIV infection was lower in Accra (0.2%)–5.1% in Wa, with an overall prevalence of 2.9%. The total prevalence of syphilis was 6.8%, with the least prevalence in Tamale (1.2%) and the highest in Nkwanta in the Oti region (13.5%). In Ghana, HBV prevalence ranges from 2.5% to 16.7%,^34^ suggesting the infection remains a public health threat even in the available potent preventable HBV vaccine era. HCV prevalence across Ghana has been reported to be 0.2%–8.4%,^34^ whereas that of HIV is from <1% to 2.8%. Worryingly, the prevalence of HIV in the Upper West region is higher than the upper limit of the national value. This poses a significant risk of blood transfusion in the region. The prevalence observed in this study could be even higher, partly due to the method of screening employed. It has been established that techniques such as the enzyme‐linked immunosorbent assay and the polymerase chain reaction are more sensitive and specific and help in preventing occult infections due to blood transfusion.^35^, ^36^ However, the one‐step immune‐chromatographic assay technique was used for HBV, HCV, HIV, and *T. pallidum* screening, which poses a risk to blood recipients.

Considering the four study sites selected across Ghana, the prevalence of TTIs were varied and diverse. The region with the highest proportion of TTIs was the Upper West (56.0%), followed by the Oti region (31.2%). Specifically, the study revealed that blood donors in the Upper West region dominated in prevalence for all the viral‐related TTIs considered in this study (HBV, HCV, and HIV), whereas donors in the Oti Region led in syphilis. This study did not capture the reasons for these variations. However, as these two regions are rural in nature compared with the others, it could be attributed to factors of rural environment as reports suggests high prevalence of TTIs among rural dwellers.^37^, ^38^, ^39^ The relatively high prevalence of TTIs observed in the Upper West region signifies a high risk of blood transfusion‐associated infections, which calls for stricter blood screening guidelines. The variation suggests the presence of unique health challenges per study area, hence the need for a tailored intervention per the region.

Blood donors were almost universally of the male sex, as about 2% of the donors were females. This is not surprising as previous studies have reported the dominance of the male sex in blood donation.^40^, ^41^ Aside cooperate bodies organizing blood donation exercises where females may be involved, it is rare to see female replacement or voluntary donors in Ghana. Possibly, there exist a misconception that females may not be considered for blood donation due to menstruation. Also, it is likely most females may not be able to donate blood due to childbirth, pregnancy, anemia, and lactation. Some studies have reported an increased risk of mortality among critically ill blood recipients from ever‐pregnant female donors.^42^, ^43^, ^44^ It is however unclear whether this could be a contributory factor to the low patronage of female in blood donation.

The period under review (2020–2022) saw a significant HBV infection with more than twice the chance of occurrence among blood donors. In 2021, HCV, HIV, and syphilis had reduced chances of infection among blood donors as against HBV. Comparatively, HBV poses a greater risk as a TTI than the other infectious agents studied. Age‐wise, HCV infection was significantly linked to donors <24 years old, whereas syphilis infection was significant in donors ages 24–27 years. It is unclear the reason for this variation; however, previous studies have reported similar patterns of infections.^37^, ^38^, ^39^

Regarding the frequency of blood donation, first‐time donors were about three times more likely to have either HBV or HCV infection. This is expected as it could be their first‐time receiving counselling and screening for blood donation. It, however, presupposes that the general population rarely screen themselves for these infections, if not on sporadic occasions such as blood donation. Frequent donors (four or more times) were significantly associated with syphilis infection but with reduced odds of occurrence. As the routes of TTIs spread are similar, this observation suggests such donors were equally at risk of contracting the other TTIs.

Generally, the recipients of transfused blood were predominantly pregnant mothers, followed by children and accident victims. Specifically, most blood recipients in the greater Accra region were pregnant mothers, then emergency ward patients. Those of the Upper West region were mostly children, then pregnant mothers and accident victims, similar in pattern to observations made in the Oti region. Also, blood groups predominately donated were O+, followed by B+, A+, and O−. These observations provide the opportunity to pre‐empt the demands of blood transfusion in the study areas.

In conclusion, this study has revealed significant disparities and a relatively high prevalence of TTIs in Ghana, specifically with regard to HBV, HCV, HIV and *T. pallidum* infection. In all, donors' age, blood donation frequency, and donors from rural areas were more associated with TTIs. The demands for blood donation vary and blood recipients were predominantly pregnant mothers, children, accident victims, and patients in emergency wards. These findings suggest TTIs remain a major health challenge requiring an enhanced and sustained policy direction to improve the safety of blood transfusion in Ghana.

## AUTHOR CONTRIBUTIONS

**Williams Walana**: Conceptualization; data curation; writing—original draft. **Ezekiel K. Vicar**: Writing—original draft; writing—review and editing. **Eugene D. Kuugbee**: Writing—original draft; writing—review and editing. **Isaac Dari**: Data curation; writing—review and editing. **Grace Bichenlib**: Data curation; writing—review and editing. **Christian N. Aneba**: Data curation; writing—review and editing. **Kwasi N. Hinneh**: Data curation; writing—review and editing. **Iddrisu B. Yabasin**: Data curation; writing—review and editing. **Koray N. Issaka**: Data curation; writing—review and editing. **Michael O. Danso**: Data curation; writing—review and editing. **Theophilus N. Amoatey**: Data curation; writing—review and editing. **Juventus B. Ziem**: Conceptualization; validation; writing—review and editing.

## CONFLICT OF INTEREST STATEMENT

The authors declare no conflict of interest.

## TRANSPARENCY STATEMENT

The lead author Williams Walana affirms that this manuscript is an honest, accurate, and transparent account of the study being reported; that no important aspects of the study have been omitted; and that any discrepancies from the study as planned (and, if relevant, registered) have been explained.

## Data Availability

The data set for this study is available with the corresponding author and will be made available upon reasonable request. The corresponding author has full access to all the data in this study and takes complete responsibility for the integrity of the data and the accuracy of the data analysis.
